# Efficiency and Productivity of Public Hospitals in Serbia Using DEA-Malmquist Model and Tobit Regression Model, 2015–2019

**DOI:** 10.3390/ijerph182312475

**Published:** 2021-11-26

**Authors:** Aleksandar Medarević, Dejana Vuković

**Affiliations:** 1 Institute of Public Health of Serbia, 11000 Belgrade, Serbia; 2 Faculty of Medicine, University of Belgrade, 11000 Belgrade, Serbia; 3 Centre-School of Public Health and Health Management, Faculty of Medicine, University of Belgrade, 11000 Belgrade, Serbia; dvukovic@med.bg.ac.rs; 4 Institute of Social Medicine, Faculty of Medicine, University of Belgrade, 11000 Belgrade, Serbia

**Keywords:** data envelopment analysis, panel data analysis, benchmarking, technical efficiency, scale efficiency, environmental factors

## Abstract

Improving productivity within health systems using limited resources is a matter of great concern. The objectives of the paper were to evaluate the productivity, efficiency, and impact of environmental factors on efficiency in Serbian hospitals from 2015–2019. Data envelopment analysis, Malmquist index and Tobit regression were applied to hospital data from this period, and public hospitals in Serbia exhibited a great variation regarding their capacity and performance. Between five and eight hospitals ran efficiently from 2015 to 2019, and the productivity of public hospitals increased whereas technical efficiency decreased in the same period. Tobit regression indicated that the proportion of elderly patients and small hospital size (below 200 beds) had a negative correlation with technical efficiency, while large hospital size (between 400 and 600 beds), the ratio of outpatient episodes to inpatient days, bed turnover rate and the bed occupation rate had a positive correlation with technical efficiency. Serbian public hospitals have considerable space for technical efficiency improvement and public action must be taken to improve resource utilization.

## 1. Introduction

A health care system is often just one of many interconnected social welfare systems in a given country [1]. Given that health care systems are not isolated from the broader social context, the specific boundaries of a given system are difficult to determine. This makes the quality of any scientific evaluation of health care highly dependent on its ability to incorporate a wide variety of influencing factors into its methodology. In the case of hospital system performance, this requires accounting for the activities of other health care institutions, legal regulations, dominant service delivery practices, the health status of the population from which the users of health services come, and general socio-cultural, socio-economic, and other social factors [2,3]. This complex network of variables affecting hospital performance raises several questions about which factors have the most influence on performance, how these missing links can be uncovered, and how a better understanding of these factors can improve decision-making. Providing answers to these questions can help decision-makers to understand the effects of specific environmental factors and managerial variables on efficiency and performance, ultimately leading to evidence-based improvements in hospital systems. In the Serbian context, much of this understanding is currently missing and needs to be examined in order to help strengthen hospital performance across the country.

Serbia is a country in Southeastern European, located in the central part of the Balkan Peninsula and classified as an upper–middle-income economy by the World Bank [4]. Since the breakup of the former Yugoslavia, Serbian society has been characterized by negative growth and net migration rates, low fertility rates, an increasing proportion of elderly people and sharp population decline [5]. Considering that, people are living longer and fewer children are born, the population will continue to age in the coming years, and this “silver tsunami” of population ageing is causing an increasing demand for social and health services [6]. This trend of rapid population ageing also means that there will be proportionately fewer people working to support increasing numbers of economically inactive individuals, with the number of unemployed and inactive residents (primarily pensioners) already exceeding the number of employed in 2020 [7].

The Serbian health system is a social health system with compulsory health insurance and broad population coverage [8]. It is organized and managed by the Ministry of Health, Provincial Secretariat for Health Care Vojvodina, and Republic Health Insurance Fund (RHIF). Contributions are the main sources of financing. The RHIF collects revenues through obligatory insurance and distributes them to health providers. Out of pocket spending has increased over the years, suggesting shortcomings in compulsory insurance schemes. The health system includes both public and private institutions. There were 41 private hospitals providing secondary level health services in 2016 [8]. However, the volume of services provided by the private health sector is small due to its limited capacities. In general, the provision of curative and preventive services is based on the activities of public health institutions organized along three levels of health care. Primary care centres, which cover the territory of one or more municipalities or towns, provide health care at the primary level through employed “chosen doctors”. A “chosen doctor” can be a doctor of medicine with no specialty, or a doctor of medicine who is a specialist in general medicine (GP), occupational medicine, paediatrics, or in gynaecology. In addition to his other duties, the “chosen doctor” refers patients to the hospital and continues treatments after discharge.

Secondary health care is organized though general and specialty hospitals. General hospitals house almost 40% of bed capacity of public hospitals in the country, providing continuous diagnostic, therapeutic, rehabilitation and emergency services for outpatients, and inpatient care when the complexity and severity of a disorder require this type of treatment. A hospital’s minimum level of services requires at least 20 beds and the provision of specialist services in the fields of internal medicine, paediatrics, general surgery, gynaecology and obstetrics [9]. These specialists services are associated with diagnostic laboratory and imaging services, as well as anaesthesiology services and hospital pharmacies. Hospitals can also expand capacity to other services if needed. Therefore, a significant number of hospitals in the districts’ administrative centers provide additional services in the fields of neurology, mental health, surgery, and internal medicine.

Specialty hospitals aim to address certain conditions or population groups, whereas general hospitals provide care for all populations and age groups, accounting for one fifth of public beds. The primary purpose of specialty hospitals is prolonged rehabilitation and long-term treatment of psychiatric disorders.

Tertiary health care is delivered in clinics, institutes, clinical hospitals, and clinical centres. Trained personnel in these institutions provides highly specialized consulting and inpatient care. In addition to health services, these institutions are often scientific-teaching bases and research centers for medical faculties [8].

The public hospital network is established across the entire territory of Serbia. The organizations of the hospital network and the Health Insurance Fund coincide with territorial organizations which consider the availability and accessibility of adequate care. At least one hospital is located in each district’s administrative centre, while small towns have independent hospitals in remote areas or if there is a significant distance between the town and the administrative centre. This encourages horizontal cooperation between hospitals within the same district. Referring some patients from local hospitals to hospitals in administrative centers for advanced treatment is the most common form of cooperation. Patient transfer in the opposite direction exists, but it is infrequent. Considering this fragmented system, Peng’s recent finding that integrative health care raises the efficiency of hospitals is also important to consider in the Serbian context [10]. Therefore, we considered it important to compare the performance differences between hospitals that have peers in the same district and sole district hospitals that have to bear the burden of inpatient care for the entire population of the district.

Hospitals account for between one-third and one-half of the total healthcare spending among OECD countries [11]. The overwhelming share of expenditure is related to inpatient care, with increasing trends in recent years. The percentage of hospital expenditure is even more significant in Serbia, comprising more than half of the RHIF’s annual spending, with workforce compensation representing the largest share of the expenditure [12]. Therefore, the performance of Serbian hospitals has been a concern of stakeholders for years, as one of the major consequences of suboptimal resources consumption is a diminished societal willingness to contribute to the system’s funding, particularly in a social health insurance system.

Since 2000, Serbia’s health system has been reformed to improve its performance, including the implementation of diagnostic-related groups (DRGs) that classify and measure inpatient activities [13]. The DRGs have been the “gold standard” for measuring inpatient operations [14]. RHIF implemented a DRGs-based hospital payment system that remunerated the variable part of total payments. That variable part represented a small fraction of total reimbursement at the time of the study, with expected increases in the years to come. The intention is to implement a more cost-cutting payment system instead of the previous system based on the purchase of work plans.

Despite a wide breadth of literature on health care and health economics, there is a noticeable lack of evidence from Serbia and other Eastern European countries on hospital efficiency regarding specific healthcare concepts, organization, and financing [15]. The possible reasons for this could be an absence of reliable data and an ingrained belief that economic principles are unsuitable for use in healthcare settings. The implementation of DRGs enabled the quantification of inpatient care, which represents the highest volume of hospital activity. Utilizing DRGs, this study aims to fill the gap in knowledge by exploring the efficiency of Serbian hospitals during periods of transition and reform, and by producing estimates of the relative efficiency of Serbian public hospitals. In order to reach that aim, we conducted a descriptive analysis of data (Section 3.1), performed data envelopment analysis (DEA) using input and output data (Section 3.2), evaluated the efficiency change between 2015 and 2019 (Section 3.3) and identified the variables that influenced hospital performance (Section 3.4). The findings are relevant for stakeholders during Serbia’s current health reforms [16].

## 2. Methodology

Our study investigated the relative efficiency of 39 Serbian hospitals based on 2019 data through a two-stage process. The first stage was concerned with the evaluation of the relative efficiency of the observed hospitals. We conducted longitudinal (panel) data analysis using the Malmquist index to support stage one findings. The second stage focused on factors that might have had an impact on efficiency scores in 2019. A Tobit regression model was employed to explore these effects and determine possible impact factors.

### 2.1. Data

This study included data from 39 general public hospitals in Serbia. Forty general hospitals operate in Serbia [17]. Novi Pazar Hospital was removed from the analysis because of a lack of data.

Ozcan named capital investment, labour, and operating expenses the three main hospital input categories [18]. In Serbia, capital investments are sporadic and could be excluded for the study. Based on Chilingerian and Sherman’s suggestion about the distinction between different types of personnel, health workers were decomposed into physicians and other health workers [19]. Physicians play a dominant role in hospital expenditure as practitioners and as managers of teams, departments, or entire hospitals. The middle-year numbers of physicians and other health workers were used for input estimation.

Outputs included case-mix adjusted discharges and outpatient episodes to cover main hospital productivity. The algorithm grouped discharges into Australia Redefined DRGs, whereas coefficients were imported from the contract Rulebook [20]. The DRG coefficient indicates the average amount of resources needed to care for patient cases under the specific DRG, relative to the average resources used for treatment cases in all DRGs.

The Serbian Institute of Public Health (IPHS) provided input and output data from routine statistics and the National hospital database. All variables utilized in DEA analysis are summarized in Table 1.

To explore the effect of external factors, we collected the data of several variables that might explain efficiency differences from 2015–2019 (Table 2). Reliability, accuracy, timeliness, and relevance were the main criteria for factor selection. Unfortunately, only a few indicators available on the community level satisfied those criteria. Since some age groups tend to be overrepresented among hospitalised patients, their share in the catchment area population might affect hospital efficiency [21].

To illustrate the issue of hospital size and its impact on efficiency, we arranged the hospitals according to the number of beds and used four groups: very large hospitals, large hospitals, medium size hospitals and small hospitals (Table 2) [22]. The group of small size hospitals consists of facilities with less than 200 beds, and it is represented by the constant in the Tobit model.

Independent variables (Z1, Z2, Z5, Z6, and Z7) were collected from the Serbian national hospital register. The population characteristics (Z3, Z4) were obtained from the Statistical Office of the Republic of Serbia as a mid-year projection of the population size of the catchment area [23]. The catchment area was congruent for districts with a sole hospital. In districts with more hospitals, the main hospital was located in the administrative centre, whereas other hospitals were located in local communities within the district. For those situations, the catchment area of the local hospital coincides with the community area, whereas all other communities are represented in the area served by the main hospital. This approach for defining the catchment area was the closest to the actual patient flow within the healthcare system following the acts of RHIF.

### 2.2. The Applicability of Data Envelopment Analysis

Techniques for efficiency measurement can be classified as parametric or non-parametric and deterministic or stochastic [14]. Parametric techniques are regression-based, presuming a specific functional form for the frontier. They are susceptible to model misspecification because the efficiency scores are sensitive to distributional assumptions. Stochastic methods are less sensitive to outliers, as part of the observed distance to the frontier can be attributed to random error. Deterministic methods do not contain a random error as they assume inefficiency as the only reason for the observed distance to the frontier. Therefore, the deterministic non-parametric approach of (DEA) is the first choice for measuring efficiency in health care, as it explores efficiency more profoundly by looking for the root of the inefficiency.

A main advantage of DEA is that it can handle a variety of inputs and outputs, which is essential when evaluating complex health systems such as hospitals. From the optimization standpoint, this method respects hospital individuality and does not require information on relative prices, allowing for more effective comparison [21]. Efficiency measures obtained via DEA can also be used in second stage (often multi-stage) analyses which can help to evaluate the efficiency predicates [18]. In such second stage analysis, the efficiency score obtained through DEA becomes the dependent variable in the post hoc regression analysis. One of the most common methods for this second stage analysis is the Tobit regression model that transforms DEA scores to be censored at “0” [24]. After the Chilingerian study, this regression method found wide application in assessing the influence of external factors on hospital productivity [25].

This combination of DEA and Tobit regression has seen significant adoption in the literature on hospital performance evaluation. Kohl et al. included 18 studies of hospital operations in their systematic review of the literature in which the Tobit regression model was applied in the second phase using transformed DEA scores as dependent variables [15]. A search of the Medline database on the 9th of November, revealed an additional 16 such papers that have been published since that systematic review was conducted [10,26,27,28,29,30,31,32,33,34,35,36,37,38,39,40]. Among the published papers, studies focused on European hospital systems were well-represented with two studies from Turkey and one study each from Ukraine, Greece, Poland, and the Netherlands [29,41,42,43,44].

As the existing studies using this methodology examine different systems in different social contexts, it is difficult to compare their results and establish specific conclusions. However, it is clear throughout the literature that broad factors such as hospital location, population density in a hospital’s catchment area, and bed occupancy ratio are associated with efficiency. Raising bed occupancy seems to increase efficiency, but only to a certain threshold, after which point it is correlated with inefficiencies, becomes a threat to the safety of patients, and jeopardizes the quality of care [45,46,47]. In every study except one, average length of hospital stay is correlated with lower level of efficiency, whereas the ratio between outpatient episodes and inpatient days has the opposite effect [30,34]. On the other hand, the effects of factors such as hospital competition, hospital size as measured by the number of beds, hospital type, the percentage of elderly patients, and the number of specific health workers per hospital bed, are contradictory and vary across studies. This variance further emphasizes the importance of conducting a DEA on hospital performance specific to the Serbian context.

### 2.3. DEA Models

DEA establishes an efficiency frontier by optimizing the ratio between weighted output(s) and weighted input(s) of each decision-making unit (DMU). The frontier represents the most pessimistic piecewise linear envelopment of the data [48,49]. The set of DMUs is supposed to contain relatively homogeneous DMUs. Therefore, we included only general hospitals in our analysis. According to Farrell, this technique compares the DMUs and assigns 1 to an efficient DMU and less than 1 to inefficient ones [48]. Farrell’s initial study was expanded by Charnes and colleagues, who suggested a new approach that uses the constant return to scale (CRS) model, which was then followed by Banker and colleagues who developed the variable return to scale (VRS) model [48,49,50].

This study considered hospitals, and each one represented a DMUi (i = 1,…,39) and produced two outputs y_j_ = (y_1i_,y_2i_) using three inputs x_j_ = (x_1i_,x_2i_,x_3i_). Two approaches can be used with the CRS and VRS models: input-oriented and output-oriented. We used the input-oriented CRS and VRS models for three main reasons. Firstly, it is easier to control the inputs in a hospital environment than the outputs. Secondly, the input-oriented approach quantifies the input reduction without changing the output quantities [14]. Thirdly, public institutions are non-profit entities seeking to provide better services, with less of a focus on financial profit.

The CRS dual linear programming model has the following mathematical formulation:

(1) $$Min\theta_{0}\;Subject\;to\overset{39}{\underset{j=1}{\sum }}\lambda_{i}x_{\mathrm{si}}\leq \theta_{o}x_{\mathrm{so}}\;s\;=1,2,3\;\overset{39}{\underset{j=1}{\sum }}\lambda_{i}y_{\mathrm{ri}}\geq y_{\mathrm{ro}}\;r\;=1,\;2\;\lambda_{i}\geq 0\;i\;=1,2,\cdots ,39$$

in which:

- θo is the efficiency score of hospital under assessment,
- x_ri_ is the quantity of input *s* used by *i*th the hospital,
- y_ri_ is the quantity of output *r* produced by *i*th hospital,
- λ denotes the dual variables that identify the benchmarks for inefficient DMUs.

The input-oriented VRS required an additional constraint for the dual CRS model. This constraint states that the sum of the lambdas is equal to one and can be written as follows:

(2) $$\overset{39}{\underset{i=1}{\sum }}\lambda_{i}=1$$

The sum of *λ* resulting from the CRS model indicates the scale under which the hospitals are operating. Thus, if we have:

∑λ>1, the inefficient hospital is operating under decreasing returns to scale (DRS),
∑λ<1, the inefficient hospital is operating under increasing returns to scale (IRS),
∑λ=1, the efficient hospital is operating at the most productive scale size.

The use of the DEA technique allows us to obtain three types of efficiencies: technical efficiency (TE) provided by the CRS model, the pure technical efficiency (PTE) provided by the VRS model, and the scale efficiency (SE) obtained from the formula:

CRS scores = VRS sores × Scale efficiency TE = PTE × SE(3)

Hence, the technical efficiency of a DMU is decomposed into pure technical efficiency and scale efficiency. This means that pure technical efficiency consists of technical efficiency not attributed to deviations from the optimal scale. There are equal or greater number of efficient DMUs in VRS than in CRS, and the assumed scores are also equal or greater [18]. The CRS frontier is prone to a lower estimate of resource utilization and greater output production than the VRS frontier. In addition, scale efficiency measures the extent to which a DMU deviates from the optimal scale, revealing the portion of inefficiency attributable to a given scale of operations. The scale efficiency allows decision makers to select the optimal amount of resources required to reach an expected production level.

### 2.4. Malmquist Total Factor Productivity Index

Before measuring productivity, we need to define it. Productivity can be represented as the ratio between outputs and inputs, in which the maximum output attainable from each input level presents the production frontier [51]. The specificity of health institutions is that they operate with a large number of inputs and outputs, many of which are difficult to express through price.

Following Malmquist’s concept, Fare et al. developed the DEA-based Malmquist total factor productivity (TFP) to include all factors of production [52,53,54]. It depends on the DEA and measures the productivity change of a specific value between time points t and t + 1. It also applies the constant return to scale over technology to assess the distance functions employed in evaluating the Malmquist TFP index. The DEA-based Malmquist TFP index is expressed using the following formula [18]:

(4) $$M_{I}^{t+1}\left(y^{t+1}{,x}^{t+1}{,y}^{t}{,x}^{t}\right)={\left[\frac{D_{I}^{t}{(y}^{t+1}{,x}^{t+1})}{D_{I}^{t}{(y}^{t}{,x}^{t})}\times \frac{D_{I}^{t+1}{(y}^{t+1}{,x}^{t+1})}{D_{I}^{t+1}{(y}^{t}{,x}^{t})}\right]}^{1/2}$$

M_I_ is the Malmquist index based on the input-oriented approach, D_I_ are the input distance functions, and x and y are inputs and outputs vectors. An input distance function indicates the amount that specific input use can be decreased while producing the same output fixed under the production possibility.

Hence, the Malmquist productivity index is divided into two elements; the first one is the technical change in efficiency (ECH) (the catch-up effect) [55]:

(5) $$\mathrm{ECH}=\frac{D_{I}^{t+1}\left(y^{t+1}{,x}^{t+1}\right)}{D_{I}^{t}\left(y^{t}{,x}^{t}\right)}$$

and the second element is technological change (TECH) (the frontier shift effect) according to the formula:

(6) $$\;\mathrm{TECH}={\left[\frac{D_{I}^{t}{(y}^{t+1}{,x}^{t+1})}{D_{I}^{t+1}{(y}^{t+1}{,x}^{t+1})}\times \frac{D_{I}^{t}{(y}^{t}{,x}^{t})}{D_{I}^{t+1}{(y}^{t}{,x}^{t})}\right]}^{1/2}$$

The change in the Malmquist productivity index (TFPCH) is the result of the multiplication of the change in technical efficiency (ECH) and technological change (TECH). If this index is greater than 1, the productivity increased between points of time t and t + 1. Otherwise, productivity decreased if TFP is less than 1, and was stagnant if it equals 1.

ECH represents the change in the technical efficiency, whereas TECH indicates the difference in technology between time points. In other words, the Malmquist index determines the contribution of diffusion and learning (efficiency change or the catching up effect) and innovation (technical change of shifts in the frontier of technology) to productivity changes [56]. The values of ECH and TECH can be interpreted based on the same principle as TFPCH.

### 2.5. Econometric Model

Hospitals’ performance is influenced by managerial skills and environmental variables beyond managerial influence. A Tobit regression model (also known as the censored model) was used to investigate the impact of those exogenous factors on efficiency scores in the second stage of the analysis. According to Hoff, the Tobit regression is sufficient to represent the second stage of DEA models compared to alternative methods, especially ordinary least squares (OLS) regression [57].

The Tobit regression allows for the identification of variables that have a significant influence on the performance of Serbian hospitals. The usual approach is to fit several different models and choose the one that gives the “best” fit under one or more statistical measures. The selected model could explain to what extent the observed factors contribute to inefficiency.

CRS-DEA efficiency scores were transformed to be left-censored at zero because the original DEA efficiency scores are right-censored. The dependent variables of the Tobit equation consist of DEA scores transformed into hospital inefficiency scores using the following formula:

(7) $$\mathrm{Transformed}\;\mathrm{DEA}\;\mathrm{Score}=\mathrm{Inefficiency}\;\mathrm{Score}=\frac{1}{\mathrm{DEA}\;\mathrm{score}}-\;1$$

As a result of the transformation, the inefficiency score was used as a dependent variable and regressed against hypothesized determinants. The interpretation of regression coefficients is the same as in OLS. However, they differ in the interpretation of the factor signs, as a negative sign indicates better efficiency, and a positive sign signifies a greater level of inefficiency. We assessed multicollinearity before created six models with the limited number of variables identified from the literature and chose the one that had the best fit as measured by Wald Chi-squared test.

## 3. Results

### 3.1. Descriptive Analysis

Table 3 reports descriptive statistics for input and output variables for the period 2015–2019. These variables were used in the evaluation of the total factor productivity of the Serbian hospitals under study. Our study used three input variables: number of physicians, the number of workers without physicians, and the number of beds. The mean number of physicians has shown slight fluctuations between 2015 and 2019, with a five-year average of 121. The mean number of workers, excluding physicians, revealed the same patterns as the previous variable, with a five-year average of 397. However, the mean number of beds increased, with a five-year average of 394 and an average increase of 1.37 per cent.

Two outputs were considered in this study: the number of inpatients with a DRG and the number of outpatients. With a five-year average of 14,574, the number of inpatients revealed fluctuations throughout the study period. The number of outpatients showed a decline in 2017; afterwards, a slight increase appeared in the last two years of the period under consideration. The five-year average of this variable was 179,460.

The data from 2019 are presented in Table 3 with related statistical characteristics of the inputs and outputs employed in the DEA models. We noticed that the median of each factor was significantly close to the mean value. Moreover, the values of standard deviations were relatively high, indicating that the resource utilization levels, and resource allocation were unbalanced.

We included several variables in the Tobit model to explore how environmental factors affect efficiency. The descriptive characteristics of those variables are summarized in Table 4. We notice there is a relatively large variation in all considered variables.

### 3.2. Results of DEA

The efficiency scores provided by the DEA model rely on the quantities of inputs and outputs. Best practice dictates that the highest efficiency consists of producing a quality of outputs using the least inputs possible. Given the limited quantities of inputs, the maximum amounts of outputs are bounded.

Table 5 presents the DEA calculations of the CRS, VRS, and SE scores for 2019. In the CRS model, we notice that 5 out of the 39 hospitals were technically efficient. These were hospitals: H1, H6, H17, H27, and H29. The findings indicated that they were efficient at the technical and scale levels. A percentage change in inputs was associated with a similar percentage change in outputs. The remaining 34 hospitals were technically inefficient. Technical efficiency scores ranged from 0.4230 to 1. The average technical efficiency score was 0.7252, which indicates that, on average, the 39 hospitals could achieve the same level of performance and the same output levels by using 27.48% fewer resources. Otherwise, hospitals needed to produce 1.3789 (=1/0.7252) times as many as outputs from the same level of inputs. Hence, an inefficient hospital had to both reduce its inputs and improve its internal practices. The CRS efficient hospitals were also efficient in pure technical and scale efficiency measures.

The variable return to scale (VRS) represents pure technical efficiency. It measures inefficiencies due to managerial underperformance only. The hospitals H11, H15, and H19 were VRS-efficient but not CRS-efficient. These hospitals were technically efficient, and the source of their inefficiency in CRS was due to environmental factors rather than technical factors. In other words, these hospitals had implemented the best practices, but their productivity differences were due to economies of scale. An enhancement in the productivity of these hospitals was possible by using increasing or decreasing returns to scale. The average VRS efficiency score was 0.7844 and the standard variation was 0.1662 (Table 6).

The scale efficiency calculated by the DEA method revealed that five hospitals (12.82% of total hospitals) were efficient and operating under constant returns to scale. Eighteen hospitals (about 46.15%) were operating under decreasing returns to scale, which means that input increases lead to less than proportional output increases. Their average scale efficiency was 0.9697. However, 16 hospitals (about 41.02%) are operating under increasing returns to scale. Their average scale efficiency was 0.8541. Increasing returns to scale is a result of positive feedback within the market to improve something already developed or to worsen an already bad situation.

The assessment of scale efficiency is crucial to address the optimal productive size of a hospital, as it suggests how resources can be allocated most effectively. Scale efficiency reveals the ability of a hospital to pinpoint the optimal productive size that provides the full advantage of economies of scale in producing maximum output per unit of input and decreasing the average unit costs of production. Concisely, hospital efficiency depends on the hospital size. We classified hospitals into four groups by bed capacity to illustrate this issue in our study. The averages of technical and scale efficiencies for each group are presented in Table 6 as follows:

**Table 6:** Averages of technical and scale efficiencies for groups in 2019.

Hospital Group	Group 1 Very Large Size	Group 2 Large Size	Group 3 Medium Size	Group 4 Small Size
Number of beds	≥600	400 ≤ beds < 600	200 ≤ beds < 400	<00
Number of hospitals in group	8	10	12	9
Technical efficiency average	0.6265	0.7713	0.7470	0.7325
Scale efficiency average	0.9510	0.9581	0.9609	0.8224

Table 6 shows that the average technical efficiency of large hospitals (Group 2) is 0.7713, above the averages in other groups. Medium and small hospitals (Groups 3 and 4) are in the second and third ranks, respectively, slightly different in their averages. Very large hospitals from Group 1 are least technically efficient, with an average of 0.6265.

As to the averages of scale efficiency, we notice that Group 3 comes first with a value of 0.9609, while the fourth group has the lowest average (0.8224). In conclusion, Groups 2 and 3 performed the best in both efficiency scores and medium and large hospitals performed better than very large and small hospitals.

Table 7 reports the efficiency reference set, or peers (also called benchmarks) for each inefficient hospital. Each pack consists of several peers against which an inefficient hospital may be benchmarked. Peers represent best practices from which inefficient hospitals may learn and even adopt policies and techniques to become efficient. For instance, inefficient H2 had two peers: H1 and H29. Therefore, H2 could adopt best practices from these peer hospitals to improve its own operations. The other inefficient hospitals had different combinations of peers. The most cited hospital as a peer was H1, which was related to 28 hospitals, while the least mentioned was H6, which was related to 10 hospitals. DEA also quantifies the amount of knowledge the hospital has to adopt from each peer in the form of a percentage of hospital contribution represented by a lambda value. The particular lambda values (λ) are available upon request, whereas their sums are displayed in Table 6. According to the lambda values, all hospitals are classified into three groups: those who operated with decreasing returns to scale and those who operated with increasing returns to scale and the most efficient which operated with constant returns to scale. The constancy of returns to scale calls into question the empirical part of Solow’s contribution [58].

We present efficiency scores for hospitals for each year in Table 7. Only 14 of 39 hospitals were on the frontier once, but only five were on the frontier more than three times. Mean and especially median values of the entire set in 2019 were below the levels seen in 2015. Panel data in the second stage will expand on this information with additional insights.

### 3.3. Results of Malmquist Index

The results of the Malmquist index are presented in Table 8, indicating that 28 hospitals improved in the TFP from 2015–2019. The number of hospitals with a Malmquist index above 1 was greatest in the final year. The overall average of the TFPCH revealed a slight improvement in productivity over the observed period.

The findings from this table indicate that nine hospitals improved their efficiency over the period 2015–2019, with the greatest gains observed between 2016 and 2017. However, the progress was not sustained in 2018 and 2019. The primary drive in efficiency was scale efficiency, whereas pure technical efficiency decreased in the observed period. These results show a technological improvement resulting from year-over-year TECH growth in 23 hospitals from 2015 to 2016 and 37 hospitals from 2018 to 2019.

### 3.4. Results of Tobit Regression Model

Variance inflation factor (VIF) was used to detect the severity of multicollinearity (Table 9). VIFs for all variables were calculated and results show that all of them to be less than 2. This indicates that multicollinearity is not a substantive concern in our study [59,60].

Table 10 presents the results of the estimation of the Tobit models. Model 6 has the higher value of the Wald Chi-squared test (169.50). In this model, we notice three statistically significant variables at 1% and two at 5%. Respectively, these variables are the ratio of output episodes to inpatient days (Z1), the proportion of people older than 65 in the catchment area (Z3), the large size hospitals (D2), the bed turnover rate (Z5), and the bed occupation rate (Z6).

At 1%, we notice that the regression coefficient regarding the ratio of outpatient episodes to inpatient days (Z1) is negative and statistically significant. One increase in this variable leads to a decrease in the inefficiency scores for 0.0185. In other words, more outpatient episodes increase efficiency. The coefficient of (Z3) was statistically and positively significant at 1%. This means that an increase of 1% of the proportion of the elderly in the catchment area increases the inefficiency score by 2.4384. The lack of competition from other hospitals in the district (Z2) correlated with greater inefficiency, although this correlation was not statistically significant. Variables related to the hospital sizes are expressed in D1, D2, and D3, representing the groups of very large, large, and medium hospitals. The constant of the model represents the fourth group. We notice that large hospitals (D2) have a negative and statistically significant correlation with inefficiency scores. Thus, that group of hospitals has a positive correlation with efficiency scores. The same result is revealed previously in Table 7.

However, the findings stipulate that very large and medium hospital size does not significantly affect inefficiency scores. As to the small hospitals, their coefficient is positive and statistically significant at 1% (represented by the constant of the model). This indicates that this group of hospitals has a positive correlation with inefficiency scores. The coefficients of the bed turnover rate (Z5) and the bed occupation rate (Z6) are negative and statistically significant at 1% and 5%, respectively. These variables impair inefficiency scores. An increase of 1% percent in (Z5) and (Z6) reduces the inefficiency scores by 0.0135 and 0.0025, respectively.

To obtained results, we used R-package deaR for DEA and panel-data analysis [61]. Additionally, Tobit regression was performed with the STATA 15 statistical package, whereas descriptive statistics were calculated using Microsoft Excel 2016 [62,63].

## 4. Discussion

As stated in the introduction, the main aim of the study was to evaluate hospitals’ performances and identify environmental factors that correlate with hospital efficiency using operational research methods.

Serbian hospitals operated at the low-efficiency level during 2015–2019, compared to most European peers [14,64]. Some peers were more inefficient than Serbia, such as in Turkey during some years and in some DEA models of Slovakian hospitals [65,66,67]. However, hospitals in the Czech Republic and Netherlands had slightly higher average efficiency, whereas hospitals in Austria and Greece performed much better [68,69,70,71]. Only five hospitals in Serbia were both technically and scale efficient in the last studied year. Three of those five hospitals were on the frontier in the starting year, suggesting minor changes among efficient DMUs. Among inefficient hospitals, almost the same number operated on either decreasing or increasing returns to scale. Our analysis intends to identify examples of good practice to allow managers at other hospitals to know how they can implement the practices of their top performing peers. Efficiency is only one characteristic of the patient-centered quality care along with timeliness, effectiveness, equity or fairness [72]. We are sure that all health professionals work for the patient’s best interests, but some are simply more efficient than others.

The most inefficient hospital is far behind the median and mean values of the complete sample. Such results might be expected from summarizing inputs and outputs data that illustrated differences in resources among hospitals. Despite all observed hospitals being general care facilities, their respective capacities to deal with local health needs differs significantly as some of them are located in remote and less populated areas and operating on a small scale [9]. Small hospitals have relatively few patients compared to their fixed operating costs, so the average cost per case tends to be higher than in larger hospitals. Moreover, they lack the resources for optimization in the face of payment changes and require time to become used to these changes. Since their efficiency did not change significantly over the observed period, there is reason to be pessimistic about their managerial capacities. To avoid leaving people in rural areas without health care, some less efficient hospitals might eventually need to be converted into nursing homes or outpatient care centres that provide specialist ambulatory care [73].

During the observed period, the productivity of Serbian hospitals increased despite a decline in efficiency. This finding is in line with similar studies in which productivity is closely related to technical improvements [74,75]. Even in studies with productivity decline, it was mostly driven by technical descent rather than efficiency changes [76,77]. In the observed period, Serbia started implementing DRGs through a pilot study and finally as a part of the reimbursement scheme. Paradoxically, productivity rather than efficiency increased throughout implementation. Increasing productivity might be explained by hospitals attempting to better position themselves before the pay-for-performance scheme is fully implemented.

The Tobit model was applied in order to evaluate external factors that can affect efficiency. Among evaluated variables, two lead to inefficiency, whereas four were associated with efficiency. The proportion of elderly in the catchment area was associated with inefficiency, which was expected [38,42]. As numbers of elderly living in an area increased, the less efficient the corresponding hospital was, and this finding is important in light of current Serbian demographic projections [78]. Elders have higher rates of prolonged hospital stay, institutional residence, and use of long-term care services. Their services consume a tremendous amount of resources and amplify hospital resources’ waste. Ageing-driven inefficiencies are another ballast that seriously jeopardizes already inefficient Serbian hospitals. The demographic situation is not better in most of the Southeast European countries [79]. There is a widespread fear that the existing health system, which was built on a model of demographic growth, will not withstand projected demand for health services [6]. Perhaps, payment regulation adjusted for unfavourable population conditions is a solution for hospitals that will not endanger their operations if ceasing operations is not an option.

Variable Z2 in the model indicated whether or not the DMU was the only hospital in the district. The hypothesis behind its inclusion was that hospitals without competition in the district would take advantage of the monopoly to achieve relative efficiency compared to hospitals with competition. However, the regression results did not support our hypothesis, and monopoly hospitals did not materialise their privileged market position. Perhaps, the absence of competition might explain this finding. A previous paper suggests that competition between public providers stimulate public hospitals to improve their efficiency [80]. Another possible reason might be better management of individual patients within hospitals in multi-hospital districts, with patients chosen for some characteristic(s) other than their needs [81]. Selective treatment of patients based on resource consumption negatively affects hospitals’ technical efficiency and is especially frequent in the prospective payment system if the reimbursement system is not sophisticated [81,82,83]. The financial benefits of choosing profitable patients are temporary, whereas consequences of delays in treatment for those who need help the most are permanent.

Our econometric study shows that hospital size is a significant factor that contributes to inefficiency in small size hospitals and efficiency in medium and large hospitals. This finding supports literature evidence that the optimum efficiency level exists in hospitals with 200–600 beds [22,71,75,84]. Small hospitals with under 200 beds cannot realize their full potential, while huge hospitals, beyond 600 beds, are also difficult to manage efficiently. The negative coefficients related to the ratio of outpatient visits to inpatient day and bed turnover indicate that an expansion of outpatient care and increasing turnover would lead to an inefficiency decrease. The reasonable utilization of beds should be associated with management realignments to facilitate patient flow. Day hospitals are part of the solution where multiple patients can use the same bed in the same shift with proper planning between procedures. Adequate care without an overnight stay will also increase bed turnover and enhance ambulatory care within existing capacities [37,85]. Patients are also interested in day hospitals that are less stressful and more comfortable, allowing them to regain everyday routine earlier [86]. Currently, day-cases are underrepresented in Serbia, but that can be gradually increased with incentives [13].

### Limitations

Our case study has limitations due to the applied method, the data, and the specific characteristics of healthcare. DEA is a non-parametric efficiency analysis that depends heavily on data accuracy under the assumption of the right level of inputs and outputs for each DMU. Researchers resort to estimation because they cannot cover all inputs and all outputs in one study. Therefore, we selected values that best reflect hospital activity with an awareness of data quality [13]. Ideally, measuring health efficiency should include the health gains of individual patients, but since data on individual health improvements is hard to collect on the national level, we chose intermediate outputs [87]. Among outputs, the most resource-intensive is inpatient care expressed through DRG coefficients not available before 2015.

Regarding resources, we have to acknowledge that “full-time equivalent” is a more accurate indicator of staff workload than the number of employees. Unfortunately, hospitals have not collected data on this indicator. Nor does the study consider the differences within the categories of physicians and other healthcare workers. The quality of labour may vary depending on individual health skills, experience, martial, and health status.

Indicators of hospital performances (LOS, BOR, BOR) were calculated using the “day-to-day method”, despite the greater accuracy of bed occupancy in hours that reflects the genuine patient occupancy of beds [88,89]. Unfortunately, we did not have such a precise measure.

The DEA’s results refer to one particular period. One may argue that the operation of a hospital in one year may be the result of a transient advantage or disadvantage. However, the panel-data analysis for the five-year interval suggests stability of hospital efficiency throughout the observed period. The traditional DEA model cannot forecast the future efficiency of DMUs or predict the efficiency of new DMUs based on the existing dataset. DEA results are relative, and at least one DMU is always fully efficient, whereas the efficiency level of other units depends on their operations and operation of other comparable counterparts [90].

## 5. Conclusions

Using the DEA method, Malmquist total factor productivity index, and the Tobit regression model, our study has empirically shown that there is a large margin for improvement in efficiency in Serbian hospitals. Even important factors that affect hospital performance but cannot be influenced, such as demographic trends, should not be out of the scope of both policy changes and shifts in hospital management strategies.

We suggest several strategies for efficiency improvements and cost reduction. Where possible, managers of inefficient hospitals should follow the example of their top-performing peers to find the proper relationship between inputs and outputs in their specific contexts. This implies greater levels of cooperation and data-sharing across the hospital system, which can be catalyzed by changes to national policy. Improving the capacities of day hospitals is another key strategy that can be implemented to enable higher patient turnover with lower costs. Managers should also consider possible mergers of small-scale hospitals in order to improve scale efficiency and realize performance gains, while accounting for potential new sources of inefficiency that may arise following such a merger [91].

Certainly, it is also important to remember that efficiency is not the ultimate goal of hospital systems, but merely a means through which the primary goal of delivering improved health outcomes can be supported. In moving towards efficient hospitals, policy-makers must remain aware of the unique challenges faced by hospitals that are isolated in their districts and must bear the majority of the burden of inpatient and outpatient care for the local population. In these instances, total efficiency (a DEA score of 1) may not be realistically achievable without a reduction in essential services and a negative impact on population health.

Future research is also needed to promote the balanced systemic development and sustainable health policy that would contribute further to hospital performance. These efforts should focus on evaluating more methods and other factors affecting the entire system’s efficiency [92]. The efficiency research is a powerful tool to improve the efficiency of hospitals because public reporting affects the behavior of healthcare professionals and organizations more than the choices of patients and caregivers [93]. The results of this work may not reflect immediately in hospital operations, but will have a net positive impact over time, especially if combined with evidence-based decision-making and consideration for unique hospital situations on the part of hospital financing administrators.

## Figures and Tables

**Table 1:** DEA input and output variables.

Inputs Variables	Description
I1	Total number of beds
I2	Total number of health workers without physicians
I3	Total number of physicians
**Output Variables**	
O1	Number of inpatient episodes weighed with DRG coefficient
O2	Number of outpatient Episodes

**Table 2:** External factors.

External Factors	Description	Coding
Z1	The ratio of outpatient episodes to inpatient days	
Z2	No other hospital in the region	1 = if it is the sole hospital in the district
		0 = if there are other hospitals in the district
Z3	The proportion of people older than 65 in the catchment area	
Z4	Proportion of infants in the catchment area	
Z5	The bed turnover rate	
Z6	The bed occupation rate	
Z7	The average length of stay	
D1	Very large hospitals (>600 beds)	1 = if the hospital has a number of beds greater than 600
		0 = otherwise
D2	Large hospitals (400 ≤ beds < 600)	1 = if the hospital has a number of beds between 400 and 600
		0 = otherwise
D3	Medium size hospitals (200 ≤ beds < 400)	1 = if the hospital has a number of beds between 200 and 400
		0 = otherwise

**Table 3:** Descriptive statistics of input and output variables, 2015–2019.

Input/ Output	Mean	Median	Maximum	Minimum	Standard Deviation
			2015		
Physicians	121	113	253	21	62.44
Workers	400	382	922	65	215.38
Beds	387	354	887	55	222.31
Inpatients with DRG	12,763	10,995	28,012	1276	7184.84
Outpatient	179,943	14,6346	380,024	16,112	101,458
Physicians	119	109	250	22	61.46
Workers	396	379	921	56	214.33
Beds	390	342	868	55	221.64
Inpatients with DRG	12,664	10,090	29,132	963	7789.70
Outpatient	186,816	161,406	371,341	17,842	105,865.20
Physicians	121	110	261	20	62.78
Workers	399	375	945	49	218.46
Beds	392	353	880	55	222.26
Inpatients with DRG	16,355	14,206	37,330	1517	10,450.44
Outpatient	173,836	148,995	328,275	16,285	94,588.07
Physicians	122	113	260	20	64.09
Workers	399	376	934	48	220.12
Beds	393	357	845	55	220.13
Inpatients with DRG	14,139	11,871	29,634	1368	8157.57
Outpatient	176,813	156,134	344,580	15,591	97,053.67
Physicians	120	110	255	16	63.07
Workers	393	369	921	45	216.10
Beds	409	365	868	64	217.43
Inpatients with DRG	16,950	16,894	44,186.00	1680	10,877.01
Outpatient	179,889	150,738	376,322.0	21,107	101,057.30

**Table 4:** The summary of the explanatory variable of Tobit model.

DMU	Z1	Z2	Z3	Z4	Z5	Z6	Z7	D1	D2	D3
H01	3.007	0	0.236	0.009	52.358	74.825	5.216	0	0	0
H02	2.333	0	0.212	0.005	51.553	76.480	5.415	0	0	0
H03	1.684	0	0.206	0.008	30.408	66.133	7.938	0	0	1
H04	2.829	0	0.226	0.009	46.499	59.338	4.658	0	1	0
H05	1.505	0	0.267	0.006	40.110	89.797	8.171	0	1	0
H06	2.890	0	0.233	0.009	43.147	78.426	6.634	0	0	0
H07	3.381	0	0.220	0.009	29.169	46.589	5.830	0	0	1
H08	1.593	0	0.206	0.008	58.652	58.667	3.651	0	0	1
H09	1.099	0	0.283	0.007	35.038	77.193	8.041	0	0	0
H10	3.589	0	0.312	0.006	30.440	44.220	5.302	0	0	0
H11	2.730	0	0.203	0.010	62.265	66.368	3.891	0	1	0
H12	1.756	1	0.239	0.007	44.046	70.901	5.875	1	0	0
H13	1.931	1	0.214	0.008	30.409	46.185	5.544	1	0	0
H14	2.338	0	0.203	0.009	51.453	58.712	4.165	0	1	0
H15	2.231	0	0.229	0.007	26.250	44.435	6.179	0	0	0
H16	2.280	0	0.298	0.006	27.962	51.014	6.659	0	0	1
H17	2.471	0	0.203	0.008	66.948	50.595	2.758	1	0	0
H18	3.968	0	0.223	0.007	43.168	48.638	4.112	0	0	1
H19	2.432	0	0.263	0.007	59.587	46.589	2.854	0	0	0
H20	1.616	1	0.258	0.007	64.705	63.834	3.601	0	0	1
H21	2.131	0	0.243	0.008	33.879	49.890	5.375	0	1	0
H22	1.972	0	0.242	0.007	31.600	59.487	6.871	0	0	0
H23	1.467	0	0.195	0.010	36.227	73.496	7.405	0	0	0
H24	1.845	1	0.230	0.009	34.946	64.599	6.747	0	0	1
H25	1.066	0	0.206	0.009	36.228	116.666	11.754	0	1	0
H26	1.386	0	0.211	0.008	38.796	55.184	5.192	0	0	1
H27	3.925	0	0.204	0.009	31.115	55.346	6.492	0	0	1
H28	1.606	0	0.235	0.006	38.880	84.011	7.887	0	0	1
H29	2.258	1	0.201	0.009	75.647	65.982	3.184	0	1	0
H30	1.342	1	0.221	0.008	23.079	56.569	8.946	1	0	0
H31	1.774	1	0.198	0.009	41.531	57.691	5.070	1	0	0
H32	3.842	0	0.195	0.009	5.930	8.383	5.160	1	0	0
H33	1.663	0	0.219	0.009	32.767	54.227	6.040	1	0	0
H34	1.803	1	0.220	0.008	34.837	68.048	7.130	1	0	0
H35	2.418	0	0.153	0.010	35.580	67.276	6.901	0	1	0
H36	1.995	0	0.190	0.010	67.119	61.997	3.371	0	0	1
H37	1.669	0	0.204	0.009	41.545	80.282	7.053	0	0	1
H38	0.337	0	0.275	0.006	35.637	35.361	3.622	0	1	0
H39	3.078	1	0.206	0.009	61.937	48.620	2.865	0	1	0

**Table 5:** Efficiency scores of CRS, VRS and SE in 2019.

DMU	Efficiency Scores			Σ*λ*	Return to Scale	Reference Set (Benchmarks)			
	CRS	VRS	SE						
H01	1.0000	1.0000	1.0000	1.000	Constant				
H02	0.8757	0.9355	0.9361	0.757	Increasing	H1	H29		
H03	0.5788	0.5809	0.9964	1.066	Decreasing	H1	H6	H17	
H04	0.8335	0.9008	0.9253	2.606	Decreasing	H1	H6	H17	
H05	0.6937	0.7193	0.9644	1.784	Decreasing	H1	H17	H29	
H06	1.0000	1.0000	1.0000	1.000	Constant	H6			
H07	0.7610	0.7611	0.9999	0.899	Increasing	H6	H17	H27	
H08	0.7998	0.8616	0.9284	0.488	Increasing	H17	H29		
H09	0.5032	0.6916	0.7276	0.257	Increasing	H1	H29		
H10	0.7005	0.8097	0.8651	0.628	Increasing	H1	H6		
H11	0.9711	1.0000	0.9711	2.503	Decreasing	H1	H29		
H12	0.6841	0.7041	0.9717	1.853	Decreasing	H1	H17	H29	
H13	0.4919	0.5164	0.9526	1.704	Decreasing	H1	H17	H29	
H14	0.8044	0.8316	0.9674	1.543	Decreasing	H1	H17	H29	
H15	0.5978	1.0000	0.5978	0.148	Increasing	H1	H17		
H16	0.6116	0.6566	0.9316	0.424	Increasing	H1	H6	H17	H27
H17	1.0000	1.0000	1.0000	1.000	Constant	H17			
H18	0.9283	0.9284	0.9999	0.952	Increasing	H1	H6	H17	H27
H19	0.7877	1.0000	0.7877	0.198	Increasing	H29			
H20	0.8554	0.9130	0.9368	0.496	Increasing	H29			
H21	0.5859	0.6049	0.9686	1.625	Decreasing	H1	H6	H17	
H22	0.5568	0.7902	0.7046	0.384	Increasing	H1	H29		
H23	0.5710	0.7293	0.7830	0.402	Increasing	H1	H29		
H24	0.6150	0.6200	0.9920	1.192	Decreasing	H1	H6	H17	
H25	0.6449	0.6773	0.9522	1.992	Decreasing	H1	H6	H17	
H26	0.5402	0.6522	0.8283	0.271	Increasing	H1	H17	H29	
H27	1.0000	1.0000	1.0000	1.000	Constant	H27			
H28	0.6864	0.6904	0.9942	1.162	Decreasing	H1	H17	H29	
H29	1.0000	1.0000	1.0000	1.000	Constant	H29			
H30	0.4230	0.4347	0.9732	1.326	Decreasing	H1	H6	H17	H27
H31	0.6190	0.6268	0.9876	1.374	Decreasing	H1	H17	H29	
H32	0.6528	0.8069	0.8090	0.262	Increasing	H17			
H33	0.5172	0.5314	0.9733	1.819	Decreasing	H1	H17	H29	
H34	0.6240	0.6634	0.9406	2.401	Decreasing	H1	H6	H17	
H35	0.7885	0.8321	0.9476	2.195	Decreasing	H1	H17	H27	
H36	0.8954	0.9664	0.9266	0.474	Increasing	H17	H29		
H37	0.6928	0.6945	0.9975	1.045	Decreasing	H1	H17	H29	
H38	0.4905	0.5426	0.9039	0.408	Increasing	H17	H29		
H39	0.9009	0.9189	0.9804	1.692	Decreasing	H1	H17	H29	
Mean	0.7252	0.7844	0.9262						
Median	0.6928	0.7902	0.9644						
Maximum	1.0000	1.0000	1.0000						
Minimum	0.4230	0.4347	0.5978						
Standard Deviation	0.1711	0.1662	0.0950						

**Table 7:** The efficiency of general hospitals under constant return to scale, 2015–2019.

DMU	Efficiency Scores (CRS)					Number of Times on the Frontier
	2015	2016	2017	2018	2019	
H01	1.0000	1.0000	1.0000	1.0000	1.0000	5
H02	0.8609 †	0.8171 †	0.9483 †	0.8566 †	0.8757 †	
H03	0.6710 ‡	0.5200 †	0.6061 †	0.6387 †	0.5788 ‡	
H04	0.9892 ‡	0.9467 ‡	1.0000	0.8697 ‡	0.8335 ‡	1
H05	0.8847 ‡	0.9104 ‡	0.7989 ‡	0.6869 †	0.6937 ‡	
H06	1.0000	1.0000	1.0000	0.9949	1.0000	4
H07	0.8937 ‡	0.8655 ‡	1.0000	0.7976 ‡	0.7610 †	1
H08	0.8387 †	0.7920 †	0.8342 †	1.0000	0.7998 †	1
H09	0.6207 †	0.5978 †	0.4597 †	0.5405 †	0.5032 †	
H10	0.8094 †	0.5673 †	0.7193 †	0.6834 †	0.7005 †	
H11	0.9636 ‡	0.9703 ‡	0.8758 ‡	0.8404 ‡	0.9711 ‡	
H12	0.8590 ‡	1.0000	0.8286 ‡	0.8046 ‡	0.6841 ‡	1
H13	0.6788 ‡	0.7282 ‡	0.8021 ‡	0.6283 ‡	0.4919 ‡	
H14	0.8924 ‡	0.9118 ‡	0.8571 ‡	0.8816 †	0.8044 ‡	
H15	0.4250 †	0.4168 †	0.5696 †	0.5120 †	0.5978 †	
H16	0.6529 †	0.5120 †	0.6622 †	0.6916 †	0.6117 †	
H17	0.7614 ‡	0.8100 ‡	0.7845 ‡	0.7383 ‡	1.0000	1
H18	1.0000	1.0000	1.0000	1.0000	0.9283 †	4
H19	0.6506 †	0.5587 †	0.5787 †	0.8754 †	0.7877 †	
H20	0.6372 †	0.6950 †	0.6883 †	0.6357 ‡	0.8554 †	
H21	0.7878 ‡	0.8121 ‡	0.8194 ‡	0.6458 †	0.5859 ‡	
H22	0.5813 †	0.5952 †	0.5410 †	0.5941 †	0.5568 †	
H23	0.7324 †	0.7477 †	0.7354 †	0.6090 †	0.5710 †	
H24	0.7942 ‡	0.7042 ‡	0.7619 ‡	0.6534 †	0.6150 ‡	
H25	0.8819 ‡	0.8364 ‡	0.8639 ‡	0.7498 ‡	0.6449 ‡	
H26	0.8947 †	0.9689 †	0.9546 †	0.7677 †	0.5402 †	
H27	1.0000	0.8351 ‡	1.0000	1.0000	1.0000	4
H28	0.7522 ‡	1.0000	0.7759 ‡	0.7087 †	0.6864 ‡	1
H29	0.9149 ‡	1.0000	1.0000	1.0000	1.0000	4
H30	0.7576 †	0.7841 †	0.8742 ‡	0.6742 ‡	0.4230 ‡	
H31	1.0000	1.0000	0.9858 ‡	0.7607 ‡	0.6190 ‡	2
H32	1.0000	0.9215 †	0.9002 †	0.7568 †	0.6528 †	1
H33	0.5627 ‡	0.6035 ‡	0.6748 ‡	0.6207 ‡	0.5172 ‡	
H34	0.7433 ‡	0.7831 ‡	0.8782 ‡	0.6837 ‡	0.6240 ‡	
H35	0.9633 ‡	0.8248 ‡	0.9432 ‡	0.8768 ‡	0.7885 ‡	
H36	0.5815 †	0.6057 †	1.0000	1.0000	0.8954 †	2
H37	0.7305 †	0.7879 †	0.6297 †	0.7137 †	0.6928 ‡	
H38	0.7414 ‡	0.7510 †	0.6762 †	0.6281 †	0.4905 †	
H39	0.8460 ‡	0.8598 ‡	0.8752 ‡	0.7652 †	0.9009 ‡	
Mean	0.8040	0.7959	0.8180	0.7663	0.7252	
Median	0.8094	0.8121	0.8342	0.7498	0.6928	
Maximum	1.0000	1.0000	1.0000	1.0000	1.0000	
Minimum	0.4250	0.4168	0.4597	0.5120	0.4230	
Standard Deviation	0.1469	0.1618	0.1500	0.1410	0.1689	

Note: † = increasing return to scale, ‡ = decreasing return to scale.

**Table 8:** The average Malmquist index, frontier shift and efficiency changes over the period 2015–2019.

DMU	Malmquist Index [TFPCH]	Frontier Shift (TECH)	Efficiency Change [ECH]	Pure Efficiency Change [PECH]	Scale Efficiency Change [SECH]
H01	1.027	1.027	1.000	1.000	1.000
H02	1.044	1.040	1.004	1.013	0.991
H03	0.989	1.027	0.964	0.964	1.000
H04	1.011	1.056	0.958	0.974	0.983
H05	1.000	1.063	0.941	0.933	1.009
H06	1.030	1.030	1.000	1.000	1.000
H07	0.978	1.018	0.961	0.956	1.004
H08	1.126	1.140	0.988	1.003	0.985
H09	1.034	1.089	0.949	0.959	0.989
H10	0.956	0.991	0.965	0.967	0.998
H11	1.065	1.062	1.002	1.000	1.002
H12	1.043	1.104	0.945	0.916	1.031
H13	0.988	1.071	0.923	0.881	1.047
H14	1.050	1.078	0.974	0.975	1.000
H15	1.180	1.083	1.089	1.000	1.089
H16	1.040	1.057	0.984	0.980	1.004
H17	1.244	1.162	1.071	1.055	1.015
H18	0.951	0.969	0.982	0.982	1.000
H19	1.111	1.059	1.049	1.051	0.998
H20	1.157	1.075	1.076	1.093	0.984
H21	1.020	1.098	0.929	0.922	1.007
H22	1.003	1.014	0.989	0.990	0.999
H23	0.988	1.051	0.940	0.974	0.964
H24	0.992	1.057	0.938	0.935	1.003
H25	0.983	1.063	0.925	0.920	1.005
H26	1.023	1.161	0.882	0.910	0.968
H27	1.015	1.015	1.000	1.000	1.000
H28	1.028	1.052	0.977	0.977	1.000
H29	1.009	1.167	0.864	0.870	0.993
H30	1.140	1.115	1.022	1.000	1.022
H31	1.023	1.153	0.887	0.890	0.997
H32	0.946	1.052	0.899	0.948	0.948
H33	1.065	1.088	0.979	0.967	1.012
H34	1.027	1.073	0.957	0.950	1.008
H35	0.988	1.038	0.951	0.957	0.994
H36	1.221	1.096	1.114	1.119	0.996
H37	1.048	1.062	0.987	0.981	1.006
H38	1.013	1.124	0.902	0.919	0.982
H39	1.099	1.082	1.016	1.004	1.011
2015–2016	1.015	1.030	0.985	0.989	0.997
2016–2017	1.099	1.065	1.032	1.013	1.019
2017–2018	0.952	1.014	0.939	0.950	0.988
2018–2019	1.103	1.178	0.936	0.936	1.001
2015–2019	1.042	1.072	0.973	0.972	1.001

**Table 9:** The values of variance inflation factor for examined environmental factors.

Variable	Z1	Z2	Z3	Z4	Z5	Z6	Z7	D1	D2	D3
Mean VIF †	1.42	1.77	1.85	1.92	1.40	1.80	1.85	1.61	1.64	1.71

Note: † VIF: variance inflation factor.

**Table 10:** Results of the estimation of Tobit model.

Variables	Model 1	Model 2	Model 3	Model 4	Model 5	Model 6
Z1	−0.0185 ***	0.0184 ***	−0.0215 ***	−0.0218 ***	−0.0219 ***	−0.0185 ***
Z2	0.0308	0.0332	…..	0.0369	0.0336	0.0346
Z3	2.7140 ***	2.9427 ***	2.6519 ***	2.5945 ***	2.4396 ***	2.4384 ***
Z4	−5.3141		…..	…..	−4.0363	−4.1911
D1	…..	…..	−0.0097	−0.0353	−0.0312	−0.0298
D2	…..	…..	−0.1155 **	−0.1231 ***	−0.1154 **	−0.1141 **
D3	…..	…..	−0.0495	−0.05844	−0.0577	−0.0596
Z5	−0.0135 ***	−0.0135 ***	−0.0164 ***	−0.0167 ***	−0.0168 ***	−0.0135 ***
Z6	−0.0026 **	−0.0025 *	….	….		−0.0025 **
Z7	….	…..	−0.0215	−0.0225	−0.02316 *	….
Constant	0.4995 ***	0.3959 ***	0.6067 ***	0.6372 ***	0.7129 ***	0.5857 ***
Observations	195	195	195	195	195	195
Number of groups	39	39	39	39	39	39
Obs. per group	5	5	5	5	5	5
Wald *X^2^*	157.78	153.72	163.97	165.12	167.55	169.50
Prob. > *X^2^*	0.0000	0.000	0.0000	0.0000	0.0000	0.0000
Log Likelihood	13.4347	12.3064	15.1307	15.4441	16.0987	16.6212

Note: ***, **, * indicate significance at 1%, 5%, and 10% respectively.

## Data Availability

Data available on request. The data presented in this study are available on request from the corresponding author.
